# Second‐line therapy with first‐ or second‐generation tyrosine kinase inhibitors in *EGFR*‐mutated non‐small cell lung cancer patients with T790M‐negative or unidentified mutation

**DOI:** 10.1111/1759-7714.13870

**Published:** 2021-02-14

**Authors:** Tadashi Nishimura, Tomohito Okano, Masahiro Naito, Soichi Iwanaka, Ayaka Ohiwa, Yasumasa Sakakura, Taro Yasuma, Hajime Fujimoto, Corina N. D'Alessandro‐Gabazza, Yasuhiro Oomoto, Tetsu Kobayashi, Esteban C. Gabazza, Hidenori Ibata

**Affiliations:** ^1^ Department of Pulmonary Medicine Mie Chuo Medical Center Tsu Japan; ^2^ Department of Pulmonary and Critical Care Medicine Mie University Graduate School of Medicine Tsu Japan; ^3^ Department of Immunology Mie University Graduate School of Medicine Tsu Japan

**Keywords:** chemotherapy, *EGFR* mutation, EGFR tyrosine kinase inhibitors, non‐small cell lung cancer, T790M negative

## Abstract

**Background:**

T790M mutation causes resistance to tyrosine kinase inhibitors (TKIs) in approximately 49% of patients with epidermal growth receptor‐mutant non‐small cell lung cancer (NSCLC). The cause of resistance in the remaining half of the cases is a minor mutation or unknown. Here, we conducted a retrospective study of epidermal growth receptor‐mutant NSCLC patients with T790M‐negative or an unidentified mutation to appraise the therapeutic response to first‐ or second‐generation tyrosine kinase inhibitors as a second‐line treatment.

**Methods:**

The study included 39 patients treated in our institution from April 2012 through March 2020 with second‐line tyrosine kinase inhibitors or chemotherapy after completing a first‐line therapy with tyrosine kinase inhibitors.

**Results:**

The patients were allocated to two groups: chemotherapy (*n* = 28) and a tyrosine kinase inhibitor (*n* = 11) groups. The median progression‐free survival (PFS) was 5.4 months in the chemotherapy group and 3.4 months in the tyrosine kinase inhibitor group (*p*‐value = 0.36), while the median overall survival (OS) was 16.1 months in the chemotherapy group and 12.8 months in the tyrosine kinase inhibitor group (*p*‐ value = 0.20). This study showed no significant difference in PFS and OS between the chemotherapy and tyrosine kinase inhibitor groups.

**Conclusions:**

These observations suggest that first‐ and second‐generation tyrosine kinase inhibitors are not recommended for second‐line treatment in epidermal growth factor receptor‐mutated NSCLC patients with T790M‐negative mutation who have received tyrosine kinase inhibitors as first‐line treatment.

## Graphical abstract

T790M mutation is the main cause of therapeutic resistance to TKI in *EGFR*‐mutant NSCLC patients. There is no effective second‐line therapy for unknown or T790M‐negative mutation patients previously treated with first‐ or second‐generation EGFR TKI. This study showed that first‐ and second‐generation TKIs are not recommended for second‐line treatment in *EGFR*‐mutated NSCLC patients with T790M‐negative or unknown mutation that have received TKIs as first‐line treatment.

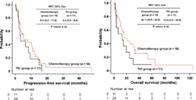

## INTRODUCTION

Among all lung adenocarcinoma cases, epidermal growth factor receptor (EGFR) mutations account for 47.9% in the Asian population and 45% in the Japanese population.^1^, ^2^ Based on clinical trials showing improvement of progression‐free survival (PFS) following treatment with first‐ and second‐generation EGFR tyrosine kinase inhibitors (TKIs), EGFR TKIs have become the first‐line treatment for *EGFR* mutation‐positive adenocarcinomas.^3^, ^4^, ^5^, ^6^ The AURA3 study showed that osimertinib, a third‐generation EGFR TKI, is effective as a second‐line treatment for T790M mutation‐positive lung adenocarcinomas.^7^ The FLAURA study showed that, as first‐line therapy, osimertinib is superior to gefitinib or erlotinib to prolong PFS and overall survival (OS).^8^ Therefore, osimertinib is currently the first therapeutic choice for *EGFR* mutation‐positive lung adenocarcinoma. However, there is no effective second‐line treatment for patients with T790M mutation‐negative or unidentified mutations who have received a first‐ or second‐generation EGFR TKI as first‐line therapy. In the FLAURA study, patients from the control (27%) and osimertinib (29%) groups received first‐ or second‐generation EGFR TKI as second‐line treatment.^9^ As patients treated with first‐ or second‐generation TKIs were long‐living, we hypothesized that the therapeutic efficacy as a second‐line treatment of first‐ or second‐generation EGFR TKIs and chemotherapy would be different. To demonstrate this hypothesis, in the present study, we compared the therapeutic efficacy between chemotherapy and first‐ or second‐generation EGFR TKIs as the second‐line treatment in *EGFR*‐mutated non‐small cell lung cancer (NSCLC) patients with T790M‐negative or unknown mutation.

## METHODS

### Patients

**FIGURE 1:**
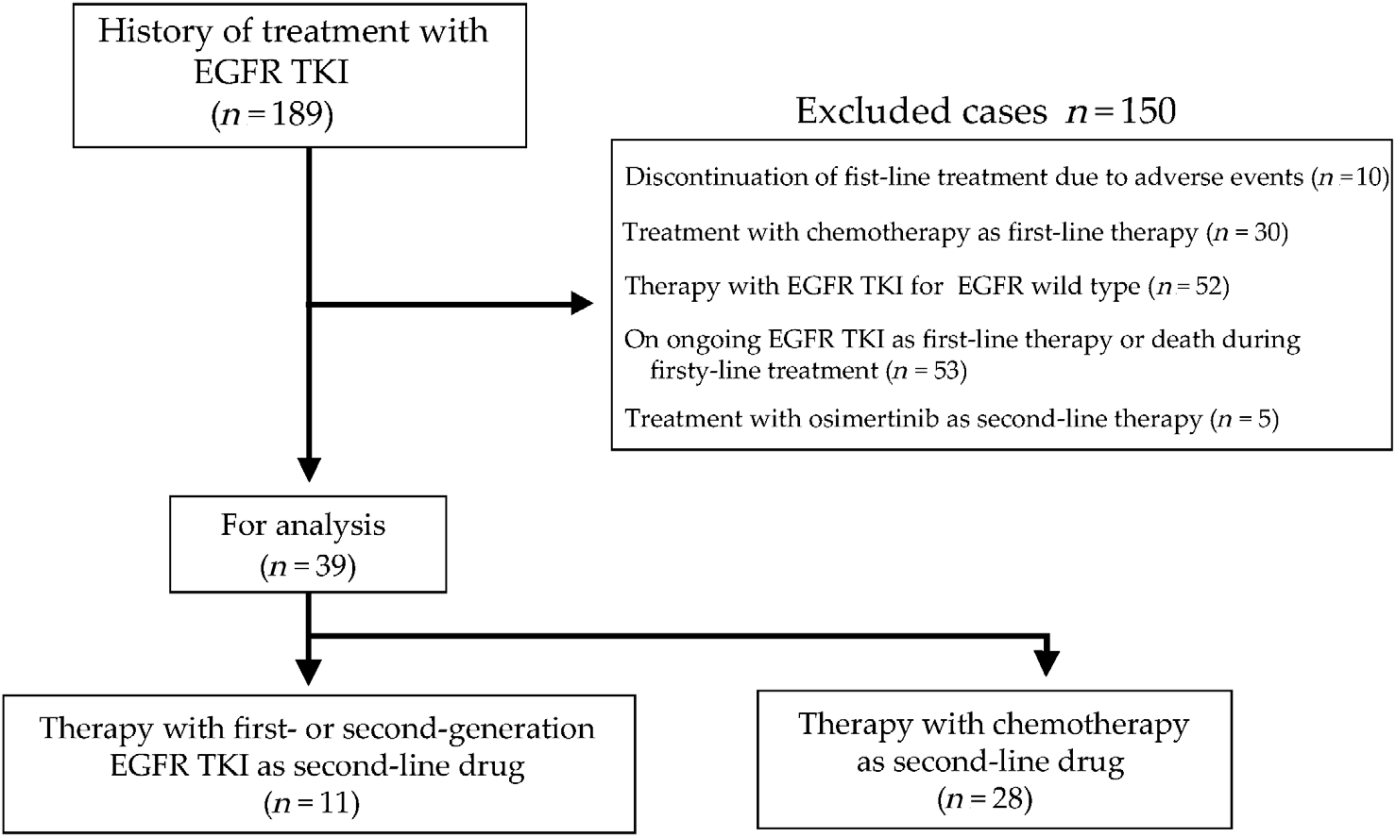
Study flow chart. A total of 39 patients out of 189 patients with a history of tyrosine kinase treatment were included in the study. EGFR, epidermal growth factor receptor, TKI, tyrosine kinase inhibitor

We retrospectively conducted this study using data retrieved from electronic medical records in our institution from April 2012 through March 2020. The study included 39 patients treated with second‐line tyrosine kinase inhibitors or chemotherapy after completing a first‐line therapy with TKIs (Figure 1). The total number of patients treated with EGFR‐TKI during the study period was 189. We excluded patients in which first‐line therapy had been discontinued because of adverse events (*n* = 10), patients treated with chemotherapy as first‐line therapy (*n* = 30), with EGFR TKIs in the presence of *EGFR* wild‐type (*n* = 52), with osimertinib as second‐line therapy (*n* = 5), or patients under ongoing EGFR TKI therapy (*n* = 53) (Figure 1). The patients were allocated into two groups: chemotherapy (*n* = 28) and tyrosine kinase inhibitor (*n* = 11) groups. The reasons for treating patients with tyrosine kinase inhibitors were as follows: five patients refused treatment with chemotherapy, four had poor performance status, one had a metastatic tumor in the brain, and one was an elderly patient.

### Statistical analysis

Continuous variables were assessed using the Mann–Whitney U test and categorical variables using Fisher's test. The Kaplan–Meier method and the log‐rank test were used for comparing survival. Multivariate analysis was performed using the Cox proportional hazards regression analysis. The Response Evaluation Criteria in Solid Tumors (RECIST) version 1.1 was used to determine efficacy. OS was defined as the time from the initial day of the second‐line treatment to death. *p* < 0.05 was considered statistically significant. We performed all statistical analyses using the EZR (Saitama Medical Center, Jichi Medical University, Saitama, Japan), a graphical user interface for R (The R Foundation for Statistical Computing, Vienna, Austria); this is a modified version of the R commander designed to add statistical functions frequently used in biostatistics.^10^

## RESULTS

### Response rate and survival

**FIGURE 2:**
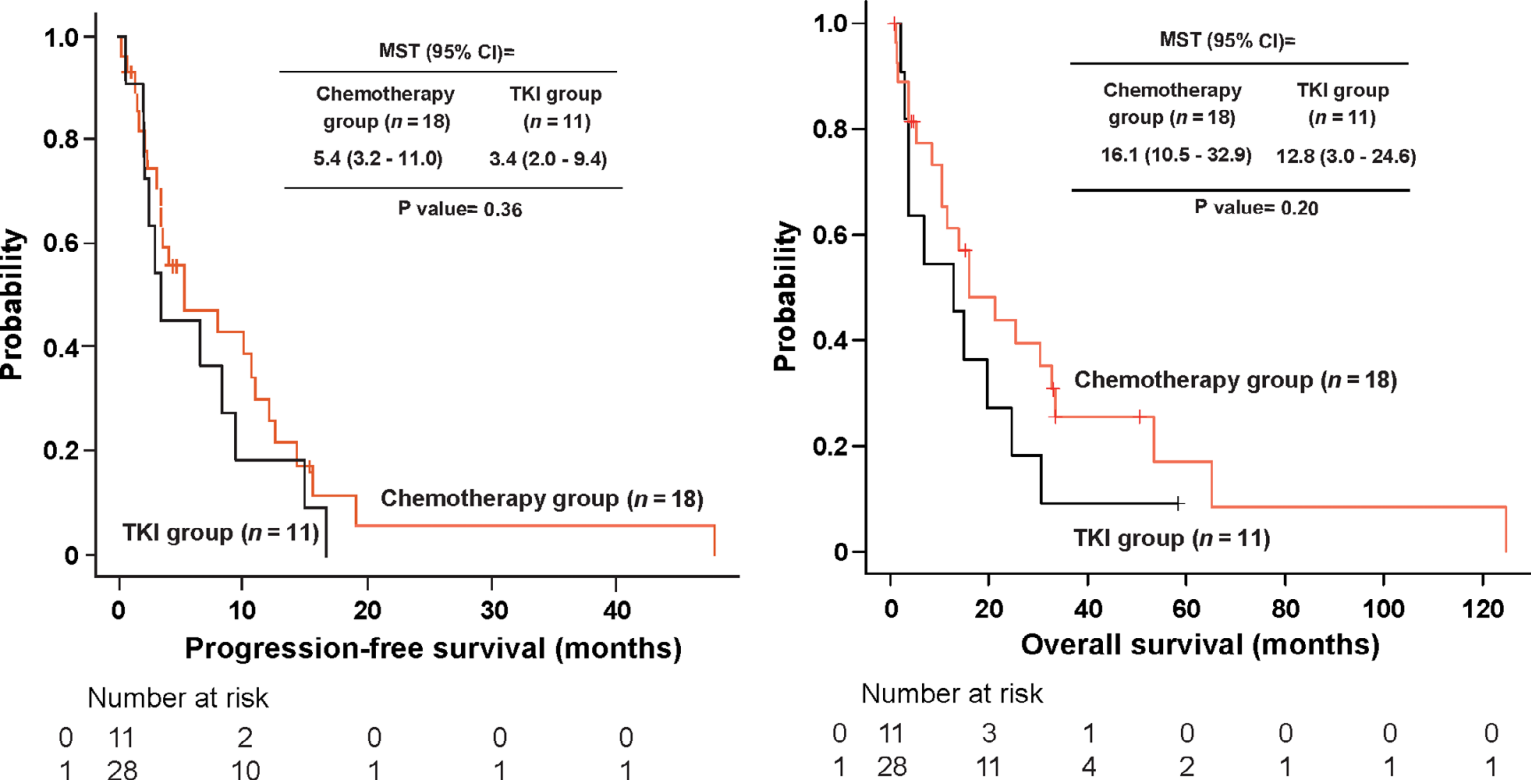
Progression‐free survival (PFS) and overall survival (OS) in each group of patients. The median PFS was 5.4 months in the chemotherapy group and 3.4 months in the tyrosine kinase inhibitor (TKI) group. The median survival time was 16.1 months in the chemotherapy group and 12.8 months in the TKI group. CI, confidence interval; MST, median survival time; PFS, progression‐free survival; OS, overall survival

In the study, we included 39 out of 189 patients with a history of treatment with TKIs (Figure 1). Tables 1 and 2 show the patient characteristics. The chemotherapy group included 28 patients (71.8%), whereas the TKI (28.2%) group included 11 patients. The Eastern Cooperative Oncology Group performance status (ECOG PS) at the start of first‐line treatment was significantly better in the chemotherapy group (*p* = 0.04) than in the TKI group. However, there was no bias in other patient backgrounds. The overall response rate was 46.4% (95% confidence interval [CI]: 27.5%–66.1%) in the chemotherapy group and 36.4% (95% CI: 10.9%–69.2%) in the TKI group (*p* = 0.725). The disease control rate was 71.4% (95% CI: 51.3%–86.8%) in the chemotherapy group and 54.5% (95% CI: 23.4%–83.3%) in the TKI group (*p* = 0.45). The median PFS was 5.4 months (95% CI: 3.2–11.0 months) in the chemotherapy group and 3.4 months (95% CI: 2.0–9.4 months) in the TKI group (Figure 2). The median survival time was 16.1 months (95% CI: 10.5–32.9 months) in the chemotherapy group and 12.8 months (95% CI: 3.0–24.6 months) in the TKI group (Figure 2).

**TABLE 1:** Patient characteristics during first‐line therapy

Number of patients	Group	Chemotherapy group (%)	TKI group (%)	*p*‐values
		*n* = 28	*n* = 11	
Median age		69.0	68.0	0.754
Gender	Male	8 (28.6)	5 (45.5)	0.453
	Female	20 (71.4)	6 (54.5)	
*EGFR* mutation	Ex19del	17 (60.7)	5 (45.5)	0.62
	Ex21.L858R	10 (35.7)	6 (54.5)	
	Ex18	1 (3.6)	0 (0.0)	
PD‐L1 status	<1%	4 (14.3)	0 (0.0)	0.35
	1%–49%	3 (10.7)	1 (9.1)	
	>50	0 (0.0)	1 (9.1)	
	Not evaluated	21 (75.0)	9 (81.8)	
ECOG PS at first‐line	0	20 (71.4)	5 (45.5)	0.042
	1	6 (21.4)	2 (18.2)	
	2	1 (3.6)	4 (36.4)	
	3	1 (3.6)	0 (0.0)	
Disease stage	I	0 (0.0)	1 (9.1)	0.127
	III	1 (3.6)	2 (18.2)	
	IV	19 (67.9)	5 (45.5)	
	Recurrence	8 (28.6)	3 (27.3)	
T790M since the third‐line	Positive	4 (14.3)	2 (18.2)	1
	Negative	9 (32.1)	3 (27.3)	
	Not evaluated	15 (53.6)	6 (54.5)	
Smoking status	Non‐smoker	6 (54.5)	19 (67.9)	
	Smoker/ever smoker	5 (45.5)	9 (32.1)	
First‐line treatment	Gefitinib	16 (57.1)	9 (81.8)	0.648
	Erlotinib	5 (17.9)	1 (9.1)	
	Afatinib	4 (14.3)	1 (9.1)	
	Osimertinib	3 (10.7)	0 (0.0)	
Liver metastasis	Positive	2 (7.1)	1 (9.1)	1
	Negative	26 (92.9)	10 (90.9)	
Carcinomatous pleurisy	Positive	14 (50.0)	3 (27.3)	0.288
	Negative	14 (50.0)	8 (72.7)	
Bone metastasis	Positive	9 (32.1)	3 (27.3)	1
	Negative	19 (67.9)	8 (72.7)	
Brain metastasis	Positive	3 (10.7)	4 (36.4)	0.083
	Negative	25 (89.3)	7 (63.6)	

Abbreviations: Chemo, chemotherapy; ECOG, Eastern Cooperative Oncology Group performance status; EGFR, epidermal growth factor receptor; TKI, tyrosine kinase inhibitor.

**TABLE 2:** Patient characteristics during second‐line therapy

Factor	Group	Chemotherapy group (%)	TKI group	*p*‐value
Number of patients		*n* = 28	*n* = 11	
Median age		70.0	68.0	0.65
Second‐line treatment	Afatinib	0 (0.0)	5 (45.5)	<0.001
	Erlotinib	0 (0.0)	6 (54.5)	
	Platinum‐based chemotherapy	17 (60.7)	0 (0.0)	
	Nonplatinum based chemotherapy	11 (39.3)	0 (0.0)	
Osimertinib approved	Before	15 (53.6)	4 (36.4)	0.48
	After	13 (46.4)	7 (63.6)	
ECOG PS	0	13 (46.4)	2 (18.2)	0.288
	1	5 (17.9)	3 (27.3)	
	2	8 (28.6)	4 (36.4)	
	3	1 (3.6)	2 (18.2)	
	4	1 (3.6)	0 (0.0)	
Liver metastasis	Positive	6 (21.4)	1 (9.1)	0.649
	Negative	22 (78.6)	10 (90.9)	
Carcinomatous pleurisy	Positive	13 (46.4)	4 (36.4)	0.725
	Negative	15 (53.6)	7 (63.6)	
Bone metastasis	Positive	13 (46.4)	3 (27.3)	0.471
	Negative	15 (53.6)	8 (72.7)	
Brain metastasis	Positive	4 (14.3)	5 (45.5)	0.085
	Negative	24 (85.7)	6 (54.5)	

Abbreviations: ECOG PS, Eastern Cooperative Oncology Group performance status; TKI, tyrosine kinase inhibitor.

### Univariate and multivariate analysis

Univariate analysis showed that ECOG‐PS and metastasis in bones, liver, and brain significantly affected the PFS and OS as assessed by the log‐rank test (*p* < 0.1). Age also affected the PFS and OS. Therefore, we included seven factors in the multivariate analysis. Age (≥75 years and <75 years), ECOG PS (≥3 and <3) during first‐ and second‐line therapy, and the presence or absence of metastasis in the brain, liver, and bones (Table 3). The dependent factors that predicted the PFS (hazard ratio [HR] = 5.24; 95% CI: 1.91–14.41, *p* = 0.0013) were ECOG PS during second‐line treatment and brain metastasis (HR = 5.05 [95% CI: 1.75–14.53], *p* = 0.0027). On the other hand, the dependent factors that predicted the OS were ECOG PS during second‐line treatment (HR = 3.19 [95% CI: 1.25–8.18] *p* = 0.015) and age (HR = 2.81 [95% CI: 1.00–7.84] *p* = 0.049) (Table 3).

**TABLE 3:** Multivariate analyses for progression‐free and overall survival

	n	Progression‐free survival		Overall survival	
		HR (95% CI)	*p*‐value	HR (95% CI)	*p*‐value
Age					
<75	31	Ref	0.81	Ref	0.049
≧75	8	1.13 (0.41–3.10)		2.81 (1.00–7.84)	
ECOG PS at first‐line					
0–1	33	Ref	0.58	Ref	0.89
2–4	6	0.72 (0.22–2.35)		0.92 (0.28–3.03)	
ECOG PS at second‐line					
0–1	23	Ref	0.0027	Ref	0.015
2–4	16	5.05 (1.75–14.53)		3.19 (1.25–8.18)	
Second‐line treatment					
TKIs	11	Ref	0.90	Ref	0.57
Chemotherapy	28	1.06 (0.42–2.65)		0.74 (0.26–2.12)	
Liver metastasis					
Negative	36	Ref	0.13	Ref	0.33
Positive	3	3.45 (0.70–16.87)		0.44 (0.08–2.35)	
Bone metastasis					
Negative	27	Ref	0.47	Ref	0.050
Positive	12	5.45 (1.83–16.28)		3.06 (1.00–9.36)	
Brain metastasis					
Negative	32	Ref	0.0024	Ref	0.28
Positive	7	5.36 (1.81–15.81)		1.89 (0.60–6.00)	

Abbreviations: CI, confidence interval; ECOG PS, Eastern Cooperative Oncology Group performance status; HR, hazard ratio; TKIs, tyrosine kinase inhibitors.

## DISCUSSION

This study showed no significant difference in PFS and OS between the chemotherapy and TKI groups.

T790M mutation has been reported to cause therapeutic resistance to first‐ or second‐generation TKIs in approximately 49% of *EGFR* mutant‐associated lung adenocarcinomas.^11^ Osimertinib has been reported to be the most effective treatment approach for T790M‐positive lung adenocarcinomas.^7^ However, there is no definite therapeutic choice for T790M‐negative adenocarcinomas. The continuous use of the same TKI despite progressive disease in resistant cases has previously been reported to have shown no clinical benefit.^12^, ^13^, ^14^ The indication for different TKIs or cytotoxic drugs may be considered as therapeutic options. Indeed, several studies have reported the effectiveness of chemotherapy after the administration of TKIs.^15^, ^16^, ^17^, ^18^, ^19^, ^20^ For example, a favorable clinical response has been previously observed with the combination of platinum‐doublet and pemetrexed, or with the combination of docetaxel and ramucirumab.^19^, ^20^

The mechanism of tyrosine kinase inhibition by gefitinib, erlotinib, and afatinib is different. Therefore, an indication of an EGFR TKI different from that initially used may be another alternative option for the treatment of resistant tumors.^21^ In cases resistant to gefitinib, erlotinib used as second‐line treatment has been shown to be moderately effective, although less effective than chemotherapy.^19^, ^22^, ^23^ In previous studies, the response rate to afatinib was 8.2%, and the impact of afatinib on overall survival was reported to be significantly different from placebo in patients with progressive tumors after treatment with first‐generation TKIs.^24^, ^25^ However, patients with poor ECOG PS were not included in these previous clinical trials, and thus the results may be incomparable to those observed in the real‐world clinical setting.^23^, ^24^, ^25^ Here, we report the results observed in the real‐world clinical setting. From our findings, ECOG PS was an independent factor for PFS in the multivariate analysis. A poor ECOG PS may influence the selection of EGFR TKI for a second‐line treatment because the frequency of adverse effects is less using TKIs. However, conducting a similar prospective study is challenging because it would be against the interests of, and possibly detrimental to, patients with poor ECOG PS. A study conducted using real‐world data before the launch of osimertinib revealed that there was no difference in OS between second‐line treatments and that ECOG PS was a prognostic factor.^26^ ECOG PS improved in T790M‐positive lung cancer patients after treatment with osimertinib.^27^ However, real‐world data showed that the median OS (9 months) of patients with ECOG PS ≥2 patients treated with osimertinib as second‐line therapy was shorter than patients with ECOG PS 0 or 1.^28^

Clinical trials are currently underway to assess the efficacy of novel molecular‐targeted drugs or immune checkpoint inhibitors in *EGFR*‐mutated lung adenocarcinoma patients with T790M‐negative or with an unknown mutation.^29^ These studies may provide new strategies for treating patients with *EGFR* mutation‐associated NSCLC with T790M‐negative or unidentified mutations.

The inclusion of patients from a single‐institution, the retrospective nature of our study, and the small number of cases are limitations of our current study. Another limitation of our study is the inclusion of cases in which the T790M test was not performed (53.8%) because the patients died before osimertinib was available in Japan. Future studies should validate the results reported here in a larger population and in patients from multiple institutions.

In conclusion, the present results showed no significant difference in PFS and OS between the chemotherapy and TKI groups for second‐line treatment in *EGFR*‐mutated NSCLC patients with T790M‐negative mutation that received EGFR tyrosine kinase inhibitors as first‐line treatment.

## CONFLICT OF INTEREST

The authors declare that they have no competing interests.
